# A “Spike-Based” Grammar Underlies Directional Modification in Network Connectivity: Effect on Bursting Activity and Implications for Bio-Hybrids Systems

**DOI:** 10.1371/journal.pone.0049299

**Published:** 2012-11-08

**Authors:** Letizia Zullo, Michela Chiappalone, Sergio Martinoia, Fabio Benfenati

**Affiliations:** 1 Department of Neuroscience and Brain Technologies, Istituto Italiano di Tecnologia, Genoa, Italy; 2 Department of Informatics, Bioengineering, Robotics and System Engineering, University of Genoa, Genoa, Italy; 3 Department of Experimental Medicine, University of Genoa, Genoa, Italy; Mount Sinai School of Medicine, United States of America

## Abstract

Developed biological systems are endowed with the ability of interacting with the environment; they sense the external state and react to it by changing their own internal state. Many attempts have been made to build ‘hybrids’ with the ability of perceiving, modifying and reacting to external modifications. Investigation of the rules that govern network changes in a hybrid system may lead to finding effective methods for ‘programming’ the neural tissue toward a desired task. Here we show a new perspective in the use of cortical neuronal cultures from embryonic mouse as a working platform to study targeted synaptic modifications. Differently from the common timing-based methods applied in bio-hybrids robotics, here we evaluated the importance of endogenous spike timing in the information processing. We characterized the influence of a spike-patterned stimulus in determining changes in neuronal synchronization (connectivity strength and precision) of the evoked spiking and bursting activity in the network. We show that tailoring the stimulation pattern upon a neuronal spike timing induces the network to respond stronger and more precisely to the stimulation. Interestingly, the induced modifications are conveyed more consistently in the burst timing. This increase in strength and precision may be a key in the interaction of the network with the external world and may be used to induce directional changes in bio-hybrid systems.

## Introduction

The ability of interacting with the environment is an essential capability of evolved biological systems. Both the nervous system internal wiring and its retained plastic properties are the primary features underlying neural network modifications and endowing a system with the ability to react to the external and internal environment. For their complexity, biological structures cannot be fully translated into an artificial system. One of the most important aspects in neurorobotics is the ability of ‘extracting’ the essential information embedded in a biological system while maintaining a satisfactory level of functionality. In order to ‘use’ a neural substrate for an artificial application, hybrids where a set of neurons (slice or dissociated cultures) are coupled with a robotic system have been an ultimate choice in the field of neurorobotics [1]. A so-build artificial system will be endowed with the ability of sense and react to external modifications. Micro-electrode arrays (MEAs) have now become a valuable interfacing technique for establishing a bi-directional communication between a neuronal network and the external world [2]–[4]. In contrast with an *in vivo* system, cultures of neurons do not contain the afferent sensory inputs and efferent motor outputs. Yet neurons in culture develop patterns of activity comparable to those recorded from developing brains [5]. They have been demonstrated to retain the ability to adapt following stimulation via potentiation and/or depression [6]–[10] and maintain and generate complex activity patterns (bursting). Many recent technological advances made it possible to connect *in vitro* neuronal networks with an artificial embodiment via sensor/actuators feedbacks in a ‘real-time’ scenario. The methodologies employed so far focused on the importance of the neurons ‘spike’ element more than their collective bursting activity. Following this rationale, an exogenous electrical stimulation of predetermined characteristics of frequency, amplitude and duration has so far been the mostly common method to induce directional changes in the network activity [7], [11]. Interestingly, in both *in vivo* and *in vitro* systems, burst firing has been proved to be involved in sensory information transmission, as well as tonic spiking and it may be more informative than single spike events in sensory input coding [12]–[14].

In this study, we stimulated networks of dissociated cortical neurons by using trains of extracellular voltage-pulses, whose inter-stimulation timing was delivered offline from the actual spike-train detected at one selected electrode during the earlier spontaneous activity. Our purpose was to investigate whether the applied stimulation was able to induce changes in the spontaneous spiking and bursting patterns of the neurons. Interestingly, we found that the burst event (i.e. the first spike of a burst) is affected by a specific modality of ‘repeated’ stimulation in which the spontaneous neuronal firing timing is respected. Our final goal was to extract the principles of dynamic modifications of a neural network that can be used in a working platform of bio-hybrids devices to optimize sensory input coding strategies to improve interaction with the external environment.

## Methods

### Cell cultures

All procedures involving experimental animals were approved by the Italian Ministry of Health and Animal Care (authorization ID 227, prot.4127 March 25, 2008). When performing the experiments we minimized the potential for nociceptor activation and painlike sensations and we respected the three R (replacement, reduction and refinement) principle in accordance with the guidelines established by the European Community Council (Directive 2010/63/EU of September 22^nd^, 2010).

Mouse embryonic cortical neurons were obtained as previously described [15], [16]. Briefly, embryos were recovered from CO_2_ anaesthetized pregnant C57BL/6J mouse at embryonic day 18 (E18). Neurons were plated onto MEAs (MEA 1060 by Multichannel System, MCS, Reutlingen, Germany) of 60 TiN electrodes (30 µm in diameter, 200 µm spaced) with internal reference. Embryos were removed, microdissected and brain cortex pieces were dissociated by enzymatic digestion in Trypsin 0.125% for 20 min at 37°C and then triturated with a fire-polished Pasteur pipette. Dissociated neurons were plated onto poly-D-lysine-and laminin-coated MEAs in a 100 µl drop covering the electrode region (1000 cells/mm^2^). One hour later, cells adhered to the substrate and 1 ml of medium was added to each device. Cells were incubated with 1% Glutamax, 2% B-27 supplemented Neurobasal medium (Invitrogen) in a humidified 5% CO_2_ atmosphere at 37°C. The 50% of the medium was changed weekly. No antimitotic medium agent was used to control glial proliferation, because application of serum-free medium limits the growth of non-neuronal cells. Neurons were allowed to grow functional and structural mature networks over a period of 2–3 weeks. Mature cultures at 18–21 days in vitro were used for the experiments.

### Ethics statement

All procedures involving experimental animals were approved by the Italian Ministry of Health and Animal Care (authorization ID 227, prot.4127 March 25, 2008) and were carried out in accordance with the guidelines established by the European Community Council (Directive 2010/63/EU of September 22^nd^, 2010).

### Recordings and stimulation

**Figure 1:**
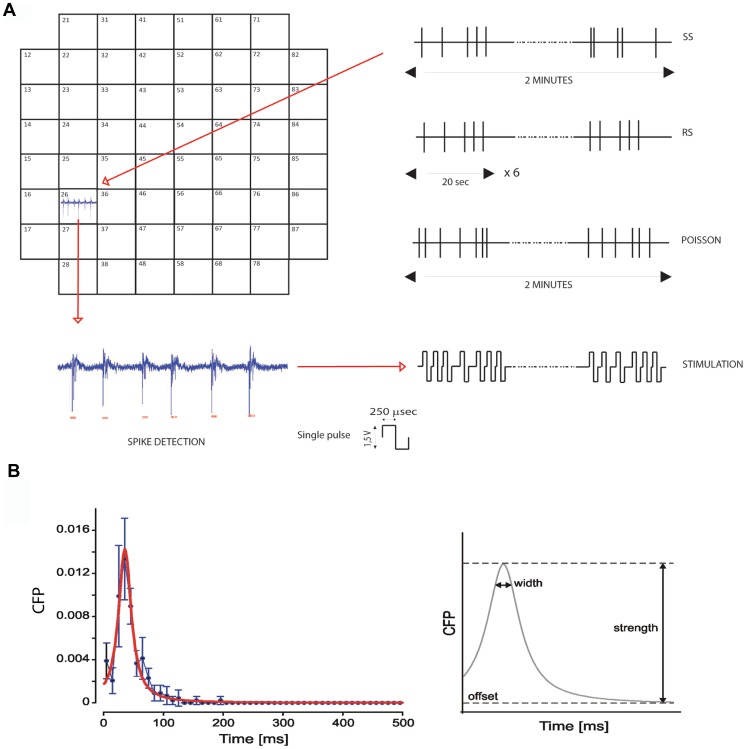
Selection and application of the stimulation sequence and network frequency-dependent modifications. A) The activity recorded from one electrode is chosen for the design of the spike pattern stimulation (see right traces). After network stabilization, its activity is extracted over a period of 2 min and a spike detection algorithm is applied to extract its spike timing pattern. A train of biphasic rectangular voltage pulses (750 mV, 250 µsec for phase, positive phase first) is built based on the spike pattern of the designed electrode and delivered through it. Three distinct stimulation protocols of the same duration (see right traces) are alternatively applied: single (SS), repetitive stimulation (RS) and Poisson stimulation (POISSON). B) Conditional Firing Probability (CFP): function and relative parameters. Left: A representative CFP histogram (black profile) and the relative fitting (red profile) between two channels during a ‘SHAM’ condition. Right: Extraction of the main parameters from the fitting of the CFP by using the equation 1 (cf. Methods section). The strength represents the maximum probability above offset, the width is computed as the amplitude of the peak at the height ‘offset+0.8 strength’, and the offset refers to background noise and unrelated background activity.

The experimental set-up was based on the MEA 60 system, consisting of: a microelectrode array, a mounting support with 60 integrated channels, a pre-and a filter amplifier (gain 1200X), a personal computer equipped with a PCI data acquisition board for real time signal monitoring and recording, an anti-vibration table and a Faraday cage. Network activity was recorded and stimulated using two commercial software, MCRack and MCStimulus (Multichannel Systems, MCS, Reutlingen, Germany) for on-line visualizations, raw data storage and stimulus sequence application. The experiments were performed outside the incubator and were maintained over the MEAs at 37°C in the culturing medium. A custom script for stimulation design on the basis of the spiking pattern was developed in MATLAB ® (The Mathworks, Natick, MA, USA). Based on its firing activity and connection with neighboring electrodes, one electrode within the network was chosen as source and sink of the stimulation pattern. A train of biphasic rectangular voltages pulses (750 mV, 250 µsec for phase, positive phase first) was delivered through the electrode of the array chosen for the design of the spike pattern stimulation (Fig. 1). This localized extracellular stimulation has previously been shown to be the most effective stimulus at any given voltage and to be effective in controlling the rhythm of bursting activity [17].

### Experimental rationale

At the developmental stage we performed our experiments (∼3^th^ week in culture), the network has reached a stable condition of maturation and synaptic connections [9], [11] and displays a rich and elaborated temporal pattern of bursting activity. Spontaneous neuronal activity was recorded for 20 minutes (pre-stimulus stage) previous to the stimulation. The stimulation phase lasted 2 minutes in all the protocols, it was delivered only once in each experiment (Fig. 1A) and was followed by additional 20 minutes of recording of the spontaneous neuronal activity (post-stimulus stage). We chose to alternatively apply three distinct stimulation protocols: single stimulation (SS), repetitive stimulation (RS) and Poisson stimulation (POISSON). The stimulus temporal pattern was built upon the spiking pattern of the spontaneous activity shown by one selected electrode. The designated stimulation electrode was one that had maintained both the recorded activity and the functional connections with the neighboring electrodes good and stable throughout the 20 minutes of pre-stimulus stage. The recording window upon which the stimulation pattern was build was chosen within the last five minutes of the pre-stimulus stage. An SS protocol is representative of a single sequence of spontaneous neuronal activity of 2 minutes. The RS protocol is composed of six repetitions of a shorter sequence of spontaneous activity (∼20 sec). The rationale behind the use of an RS protocol is that this will ‘emulate’ the repeated presentation of one stimulation pattern, as it happens during training for learning when inducing process of learning [18]. Poisson stimulation was used to double check the effect of a stimulation pattern tailored to the network spontaneous firing.

The POISSON sequence was built by producing a new distribution of pulses with same firing rate of the selected electrode of the network, but with an Inter Spike Interval histogram (ISIh) following the Poisson distribution. To do that, we developed a custom MATLAB script which also calculated the Coefficient of Variation of the output process. As expected [19], [20], the CV obtained for the sequences of stimuli in case of the POISSON experiments was 1.008±0.209 (mean ± sd).

While in the RS protocol the stimulus does not erode the fidelity of the temporal coding, this fidelity is lost in the POISSON protocol and the network undergoes a single stimulation where the frequency of firing is respected, but a randomization in the temporal pattern is introduced. A series of ‘SHAM’ experiments, in which no stimulation was delivered to the network, was performed in order to evaluate the spontaneous changes in network activity during the 40 min of the experiment.

### Data analysis and statistics

Data analysis was performed off-line by using a custom software developed in MATLAB ® named SPYCODE [21], which collects a series of tools for processing multichannel neural recordings. Briefly, data were imported in MATLAB from .mcd files (MCS format) and then spike-detected using the PTSD (Precise Timing Spike Detection) algorithm [22]. Afterwards, spike trains were analyzed by using a custom burst detection method (Pasquale et al., 2010), whose input parameters were directly estimated from the inter-spike interval distribution of each channel. Once spike and burst detection procedures were performed, several parameters describing the electrophysiological patterns could be extracted, such as the mean firing rate (MFR; spikes/sec), the mean bursting rate (MBR; bursts/min) and the inter-burst-interval (IBI; the time interval in min between two consecutive bursts). We assumed a normal distribution of spike and burst events considering the large number of output spikes available in our data sets [23], [24].

We used the cross-correlation analysis to evaluate the network functional connectivity and the effect of local stimulation on the spontaneous spiking and synchronous bursting activity of the network. Investigating the relationship between electrodes pairs gives identity to different neuronal pathways and discloses selective modifications (enhancement or reduction) occurring in the network. More specifically, a modified version of the cross-correlation algorithm named Conditional Firing Probability – CFP [24] was applied to each spike as well as to burst event trains (i.e. trains containing only the first spike of each burst). This method has been widely used in the MEA field and has been demonstrated to fit well in the context of spike train analysis [24]–[28].

In the present study, the conditional firing probability CFPij(τ) is defined by the occurrence of an event (i.e. a spike or a burst event) at electrode j at delay τ (0≤τ≤500) after that an event has been detected at electrode i, divided by the total number of events at i. To analyze the measured signals, binary arrays Xi were constructed for all recording sites i = 1, …, 60, with as many data points as the sampled signals, with Xi[n] = 1 at a detected action burst and Xi [n] = 0 elsewhere. CFPi,j[τ] is a measure related to cross-correlation Ri,j[τ] [24], [25]. If CFPi,j[τ] showed a distribution that clearly deviated from flat, electrodes i and j were considered related. CFP curves were then fitted by the following equation:
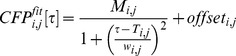
(1)where Mi,j is the maximum above offset and Ti,j is the delay at which the CFP fit function reaches its maximum value. Parameter wi,j determines the shape of the curve (the width at the 80% of the maximum above offset) and offseti,j reflects unrelated background activity. In the present study, for each pair i,j we derived the parameters M and w related to the strength (M) and the width (w, i.e. a measure of precision) of their connection (Fig. 1B). More specifically, for all the electrodes pairs showing values of M different from zero, we computed the histogram of M values. The description of the CFP in term of the strength (M) and width (w) parameters allows a higher degree of freedom than the common probability density functions and allowed us to interpret the network connectivity properties.

We then selected two ranges of data: the strong connections, related to M values between the 80^th^ and 100^th^ percentile of the distribution, and the medium connections (20^th^–80^th^ percentile). We discarded the weak connections (<20^th^ percentile). The values of w were directly obtained from those selected for M.

Statistical analysis was performed to assess the data distribution and the difference between the stimulation experiment conditions and the sham reference condition. The normal distribution of experimental data was assessed using the Kolmogorov-Smirnov normality test. All the data sets were normally distributed and Student's t-tests were performed accordingly. P values<0.05 were considered significant. Statistics on the Inter-Burst Interval distributions computed before and after stimulus delivery was performed through the paired-Wilcoxon signed rank test. All statistical analyses were carried out by using OriginPro (OriginLab Corporation, Northampton, MA, USA) and Sigma Stat (Systat Software Inc., San Jose, CA, USA).

## Results

### Modifications in network activity evoked by stimulation patterns

**Figure 2:**
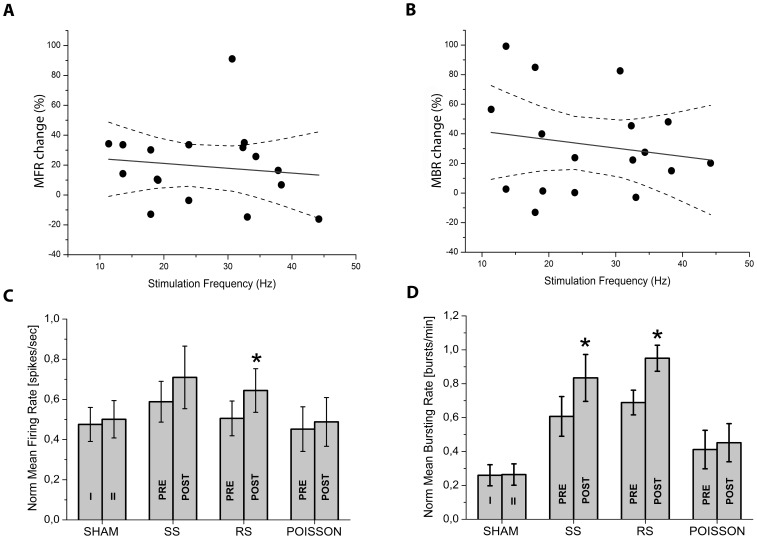
Network mean firing rate (MFR) and mean bursting rate (MBR) modifications. A, B) Variations of the network MFR (A) and MBR (B) are reported in relation to the frequency of the applied stimulation. C, D) Normalized MFR (C) and MBR (D) calculated in PRE and POST stimulus stages for the four considered experimental conditions: SHAM, SS, RS and POISSON stimulation. Only RS causes a significant increase in the level of both the firing rate and the bursting rate after stimulation. *, p<0.05; t test.

Different cultures of primary neurons have intrinsic firing pattern variability. Given that the stimulus delivered to the network retains its typical frequency and temporal pattern, in our experiments each network could have received very different stimulation sequences. We therefore checked for the influence of the stimulus frequency on the network response. We found that network spontaneous activity typically ranged from 10 to 70 Hz. We evaluated the percentage variation of the mean firing rate (MFR) and mean bursting rate (MBR) of each network in response to the applied stimulus frequency. We found that the stimulation frequency did not induce directional changes in the network MFR (Fig. 2A, B; linear regression, adj R^2^ = 0.0499; p>0.05) and MBR (linear regression, adj R^2^ = −0.0354; p>0.05).

**Figure 3:**
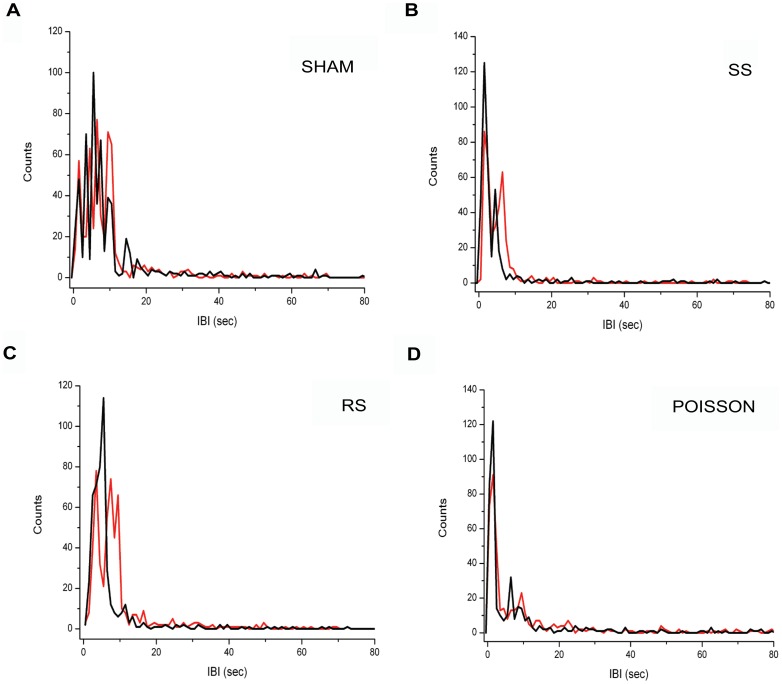
Cumulative IBI histograms. IBI histograms calculated for the SHAM (A), SS (B), RS (C) and POISSON (D) experimental groups before (red trace) and after the stimulation (black trace). While the frequency profile is not influenced by the applied stimulation in the SHAM, SS and P groups, the IBI histograms calculated for the RS experiments shows a significant shift of the peak of IBI distribution toward lower values (paired sample Wilcoxon test, p<0.05).

Twelve SHAM experiments, in which no stimulation was delivered to the network, were conducted. In these experiments, the spontaneous network activity was monitored and analyzed as for the stimulated networks to check for possible spontaneous modifications in activity during the 40 min of experiment. They were used as a ‘control’ for the stimulated networks. In these experiments we found that on a temporal scale of 40 min, both MFR and MBR tended to remain constant (Fig. 2C, D; t test, p>0.05). The bursting activity was not markedly re-organized and we did not observe any directional change in the IBI (Fig. 3A; paired sample Wilcoxon test, p>0.05).

In SS experiments (n = 8), stimulation was designed on the network firing pattern for the entire stimulus window of 2 min. No effects on the MFR nor on MBR were induced (Fig. 2C, D; t test, p>0.05). IBI values were also not influenced by the applied stimulation (Fig. 3B; paired sample Wilcoxon test, p>0.05). RS experiments (n = 9) were based on the application of a stimulus composed by six repetitions of a 20 sec single sequence respecting the firing pattern of the culture. Interestingly, a general increase in both the MFR and MBR was observed (Fig. 2C, D; t test, p<0.05) and the peak of IBI distribution was significantly shifted toward a lower value (Fig. 3C; paired sample Wilcoxon test, p<0.05). These results were consistent with previous observations in which we found that the feedback stimulation pattern needs to be repetitive over a certain time, in order to achieve modifications in a neuronal culture (*data not shown*).

In POISSON experiments (n = 11), a randomization in the spike timing pattern was introduced for the entire 2 min stimulus window, while respecting the frequency of firing. We found that both MFR and MBR did not significantly change after stimulation (Fig. 2 C, D; t test, p>0.05) and, similarly, IBI values were comparable in the two experimental stages (Fig. 3D; paired sample Wilcoxon test, p>0.05).

### Modifications in the network connectivity: cross-correlograms, connectivity strength and precision

Connectivity analysis showed that network connectivity gets reorganized in most of the cultures and depending on the applied protocol. From each experiment, we extracted the strength (M) and width (w; i.e. 1/precision) of the conditional firing probability for electrode pairs (CFP; *see Materials and Methods* and Fig. 1B). We calculated the CFP starting from both the spike trains (ST) and the burst event trains (BE). For each experimental stage, we built the distribution of the strength and width values and selected two ranges of data: the values obtained from the 20^th^ and 80^th^ percentile of the histogram (‘Perc-20’ values, relative to the medium-strength connections) and the values obtained from the 80^th^ and the 100^th^ percentile of the histogram (Perc-80’ values, relative to the strong connections).

**Figure 4:**
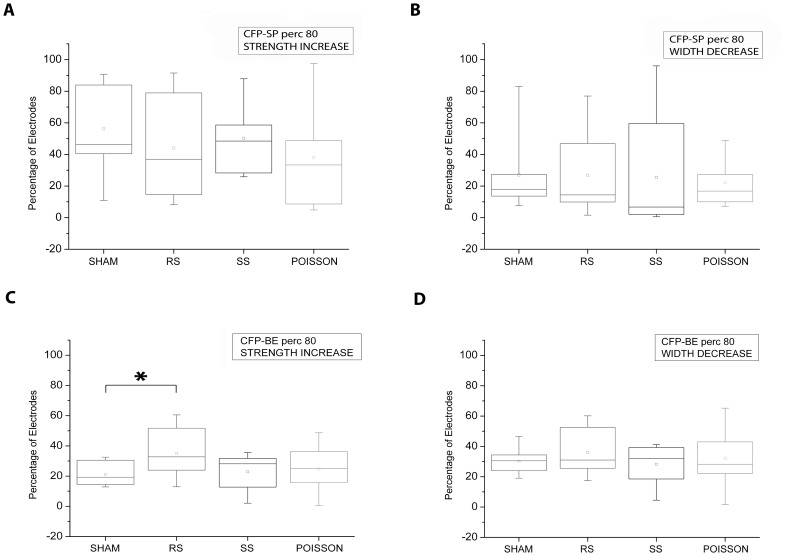
Stimulus-induced modification in the network CFP based on spike and burst events. A, B) Box plots of the percentage of electrode pairs showing a directional variation of spike train CFP (CFP-ST) strength (A) and width (B) in the post stimulus stage. C, D) Percentage of electrodes pairs showing a directional variation of burst event CFP (CFP-BE) strength (C) and width (D) in the post stimulus stage. *, p<0.05; t test.

We found that the network CFP strength and width based on 80^th^ percentile spike trains (CFP-ST) did not show directional changes in response to any of the applied protocol (Fig. 4 A, B). Interestingly the CFP strength on 80^th^ percentile calculated on burst events (CFP-BE) were indeed affected by the stimulation sequences. In particular, in RS experiments a higher number of electrode pairs showed an increased M in the post-stimulus stage if compared to the SHAM experiment (Fig. 4C; t test, p<0.05). Both Poisson and SS experiments showed no significant differences from the SHAM group (Fig. 4C t test, p>0.05). No significance difference among any of the experimental groups was revealed also in the number of electrode pairs involved in CFP width variation calculated for bursts (Fig. 4D t test, p<0.05 for each group).

We then checked if the CFP parameters tended to co-variate and therefore to be intrinsically connected one to the other. To do that, we quantified the level of connectivity modification that networks underwent in each experimental protocol (network increase and decrease in width and strength). Variations in connectivity strength can be associated with increase or decrease in width (decrease or increase of precision). Interestingly we found that increments in strength are associated either with smaller increase in width (smaller decrease of precision) or with larger decreases in width (higher increases in precision) than decrement in strength. For each experimental group, the difference between the width variations for strength increase and strength decrease was also statistically significant (*data not shown*). This can account for a general tendency toward stabilization (increase in precision) of the stronger connections versus destabilization (decrease in precision) of the weaker connections.

**Figure 5:**
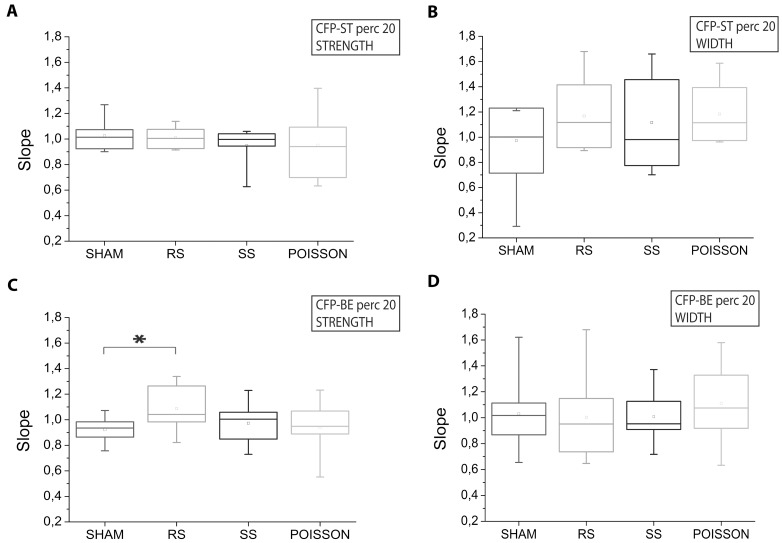
Stimulus-induced modification in the Perc-20 CFP based on spike and burst events. Box plots of the slopes resulted from the fitting procedure of the Perc-20 values of strength (A) and width (B) of the spike trains (ST) and strength (C) and width (D) of burst events (BE) before and after the application of each protocol. *, p<0.05; t test.

**Figure 6:**
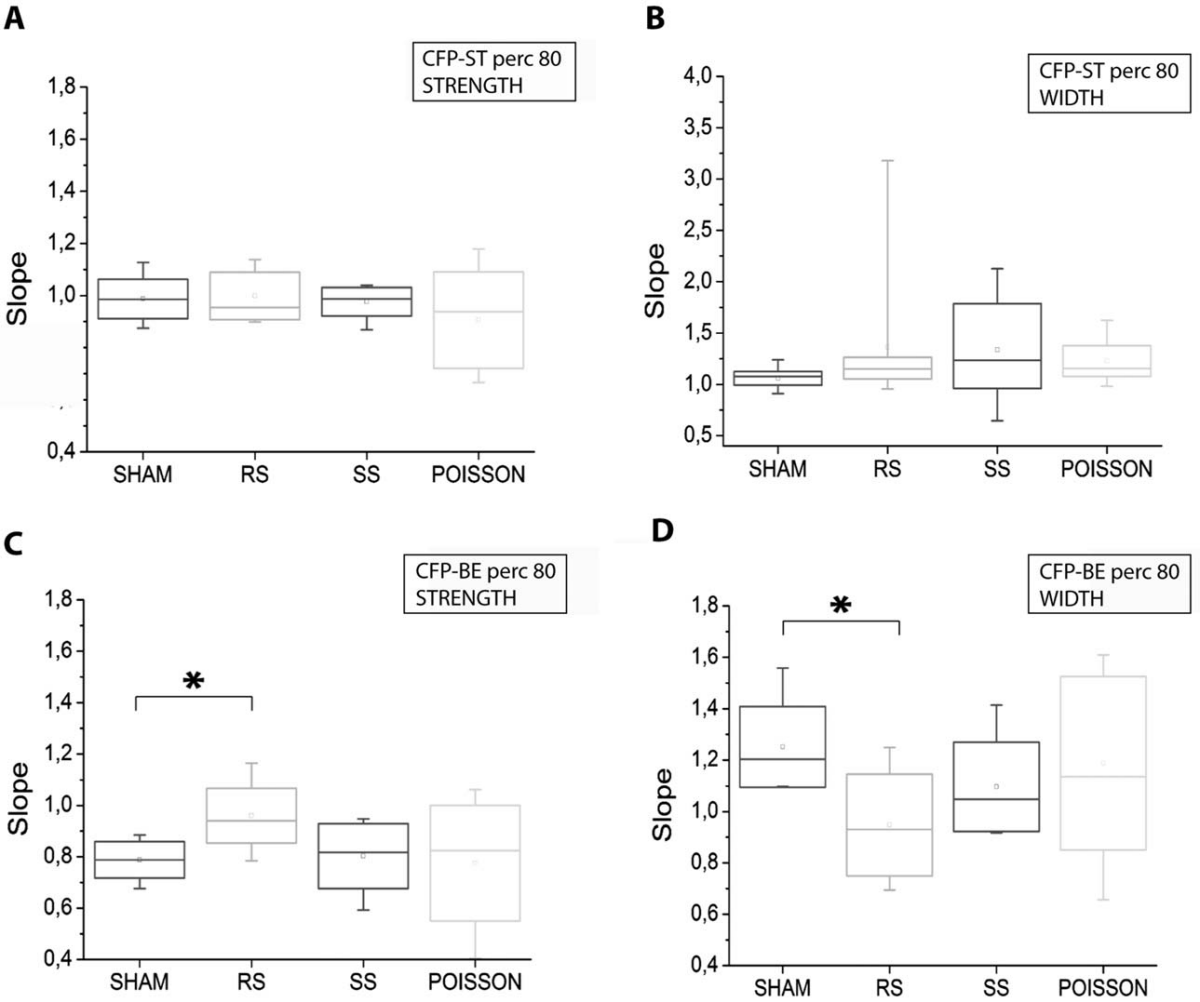
Stimulus-induced modification in the Perc-80 CFP based on spike and burst events. Box plots of the slopes resulted from the fitting procedure of the Perc-80 values of strength (A) and width (B) of the spike trains (ST) and strength (C) and width (D) of burst events (BE) before and after the application of each protocol. *, p<0.05; t test.

We then computed the tendency of the strength and width to increase, decrease or stay the same by evaluating their linear fitting before and after the application of each protocol. The slopes resulted from the fitting procedure of each experiment relative to the Perc-20 values are reported in the box plots of Fig. 5. No difference was revealed among the four experimental conditions for both strength and width of spike trains (Fig. 5 A, B). Considering only the burst-event spikes (which are a subset of the spike train), a significant difference in the CFP strength was found only between the RS and the SHAM protocol (p<0.05, t-test) (Fig. 5C, D). The ‘strongest connections’ (i.e. Perc-80 values, Fig. 6) fitting showed again no difference for the whole spike set between any of the experimental groups (Fig. 6 A, B). On the other hand, considering the burst events, a significant difference between the RS conditions and the SHAM was found for both the strength and the width parameters (p<0.05, t-test) (Fig. 6 C, D).

## Discussion and Conclusions

Nervous system plasticity is based on activity-dependent synaptic modifications such as LTP or on new forms of synaptic modification which require a more precise pattern of spiking activity such as the spike timing-dependent plasticity. In neural circuits, the spike timing-dependent plasticity mechanism may underlie processes of computation and storage of the information [29]. Many studies are now pointing toward the importance of ‘timing’ of neural activity (i.e. inter-pulse interval) for correct sensory representations and directional network modifications [30]–[33]. Precise spike timing seems also to promote *in vivo* cortical network oscillations which may contribute to the flow, representation and storage of information in neural circuits [30], [34], [35].

The methodologies employed so far in neuro-robotics focused on the importance of the ‘spike’ element of a neuron. However, *in vivo* neural circuits show an alternation of irregular spiking and high-frequency bursts. Network bursting is indeed an important aspect of neuronal activity and is naturally occurring in many brain regions. Thus it seemed reductive to focus only on the spiking, and not on the bursting properties of a network assembly.

Network bursts carry information on both rate and timing of neuronal spikes and this conveys the information expressed by the network. Recent studies are now pointing toward the importance of a ‘burst’ timing-based rule for stimulus encoding in various neural systems and various aspects of the animal behavior. Burst spiking has been proved to be a form of associative plasticity [36] which may guide processes such as reward-based reinforcement learning and goal-directed behaviors through mechanisms of burst-time dependent-plasticity [37], [38]. Interestingly, this new form of plasticity, which has been shown to be associative, input specific and reversible, obeys the same timing rule of the cue-reward learning paradigms in behaving animals [38]. In neuronal networks, precisely timed sequences of bursts of action potentials spread over the entire network are non-random, repetitive [39]–[43] and represent a general property of self-organizing networks [44], [45].

Based on that, our idea has been to apply stimulation sequences tailored on the network spontaneous activity, and therefore tailored on the single neuronal culture. Here we show that the exposure of a cortical network to a repeated stimulation pattern (RS protocol) retaining the network temporal pattern induces an increase in the network MFR and MBR, as well as substantial changes in the network IBI. This massive effect is not visible with a single delivery of the stimulation pattern. Interestingly, *in vivo* studies have shown that the repetitive exposure to an identical stimulation improves the timing precision of action potentials and promotes synchronization of cortical activity relevant to several behaviors [30]. In cultured neuronal networks, synchrony has been already suggested to be important for the functional efficacy of a network, where synchronous and asynchronous activity can enhance or reduce synaptic efficacy [10].

In order to check if and what modifications in the network synchronization occurred in response to the repeated stimulation applied, we used between pairs of active channels a cross-correlation based algorithm named CFP [46]. This method has been widely used in the MEA field and has been demonstrated to fit well in the context of spike train analysis [24]–[28]. In order to use this methodology, a normal distribution of the output spikes was assumed. This was justified by the richness in independent identically distributed random variables of our neuronal networks. Under this condition the heterogeneity of individual cells is lost and responses can be seen as the sum of identically distributed random variables [23], [24]. We found that the connectivity strength between electrode pairs gets reorganized following stimulation, independently of the applied protocol. All networks showed a general tendency toward stabilization (increase in precision) of stronger connections versus destabilization (decrease in precision) of weaker connections.

We can explain this finding by assuming that fewer constraints may limit a decrease in the connectivity precision (and therefore electrode pairs may undergo larger variation of precision), while a precision increase may be limited by an intrinsic boundary of the network. The stimulus pattern seems to play an important role in determining the ‘efficacy’ of the stimulation at the network level. It seems that the network achieves that by preferentially acting on the burst more than on spike properties. In fact RS stimulation was the only one able to induce directional changes in the network response and that these changes were mostly carried by the bursting of the network.

We also found that this stimulation increases the bursting strength and precision of the stronger connections, while only partially affects the weaker connection. This may be a mechanism exploited by the network to strengthen the connections that are more informative and probably more relevant to the overall network activity.

We think that following RS stimulation, the network reorganization points toward a selection of the connections increasing their strength and precision thus acting as a positive feedback for weakening the less reliable connections. Peculiarly, most of these modifications are based on a burst timing rule. The fact that Poisson stimulation (where the temporal structure of the neuronal activity is lost, but the overall frequency is retained) did not induce similar directional changes confirmed our hypothesis that the information brought by the stimulus is carried by its temporal structure, and not by the overall frequency of stimulation.

Interestingly, a recent study showed that the application of a ‘naturalistic’ endogenous electric field (stimulation based on the network local field potential) to an *in vitro* neocortical model system may guide network activity and have functional implications for patterning of activity sequences [47]. In conclusion we have shown that electric stimulation tailored on the network endogenous activity is able to efficiently induce modifications in the network synchronization and, in particular, affect the network bursting properties. The strongest connections are able to respond to this external modification by reinforcing their connection efficacy against the weaker connections.

Here we suggest the use of a spike timing rule to direct modification in networks of bio-hybrid devices. A so-build system will be fine-tuned on changes in the external environment and will be endowed with the ability of reacting more reliably to external modifications. This study offers a new perspective to break into the ‘grammar’ of neuronal activity and in its functional interactions with the real world.
